# Supplementary material for: Proposal of Bacillus altaicus sp. nov. Isolated from Soil in the Altai Region, Russia
Source: Int J Mol Sci. 2025 Sep 29;26(19):9517. doi: 10.3390/ijms26199517 (PMC12525529; doi:10.3390/ijms26199517)
Supplement: Supplementary file 1 [file ijms-26-09517-s001.zip › ijms-3870185-supplementary-figures.pdf]

Supporting Information for

Proposal of *Bacillus altaicus* sp. nov. isolated from soil in the Altai region, Russia

Anton E. Shikov [1 †], Maria N. Romanenko [1,2 †], Fedor M. Shmatov [1], Mikhail V. Belousov [1,2], Alexei Solovchenko [3], Olga Chivkunova [3], Grigoriy K. Savelev [1], Irina G. Kuznetsova [1], Denis S. Karlov [1], Anton A. Nizhnikov [1,2], and Kirill S. Antonets [1,2*]

[1] All-Russia Research Institute for Agricultural Microbiology, 196608 St. Petersburg, Russia
[2] Faculty of Biology, St. Petersburg State University, 199034 St. Petersburg, Russia
[3] Department of Bioengineering, Faculty of Biology, Moscow State University, Moscow 119234, Russia
* Correspondence: k.antonets@arriam.ru (K.S.A.)
† These authors contributed equally to this work.

This file includes:

Figures S1 to **S6**

Descriptions of **Tables S1** to **S11**

1. Supplementary figures

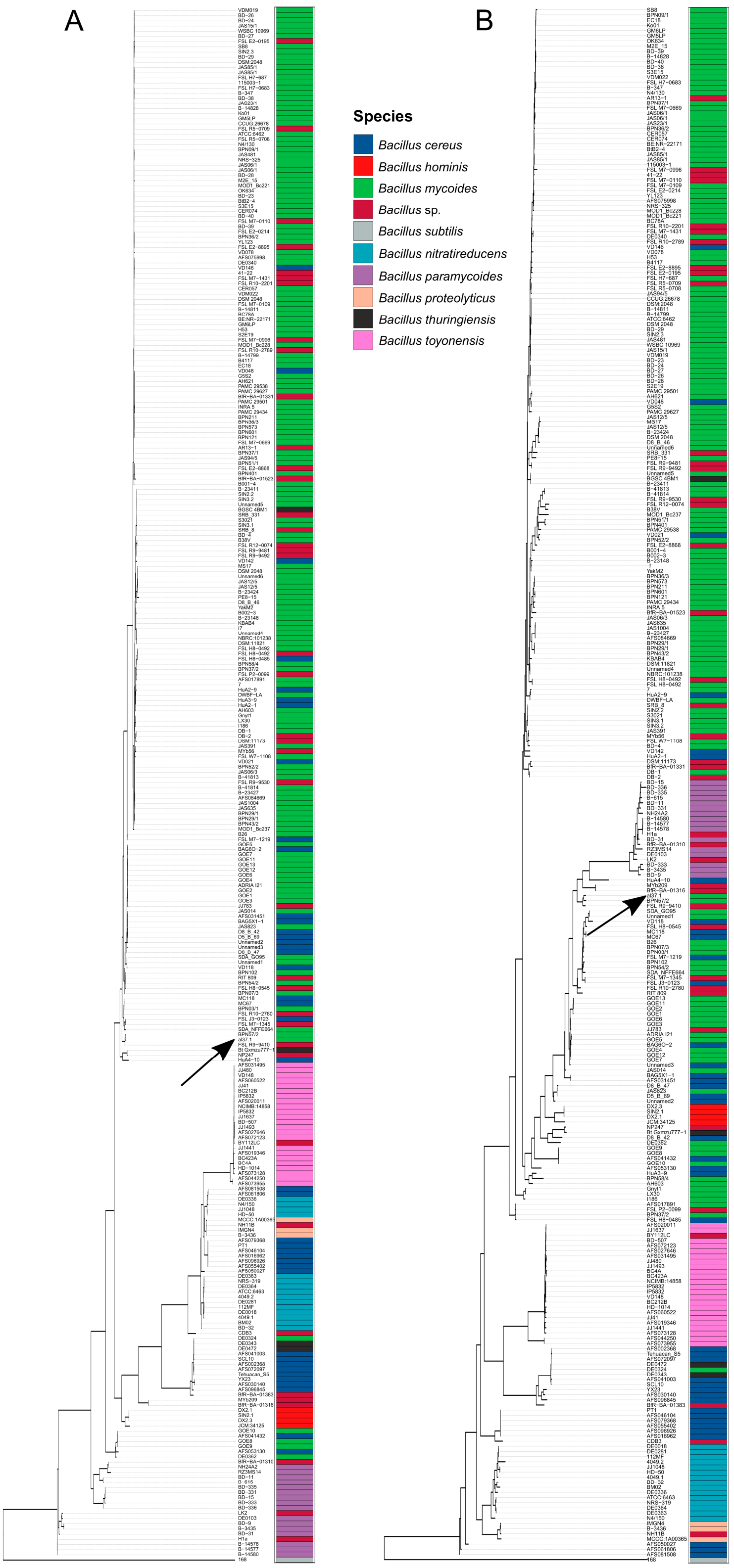

Figure S1. Phylogenetic inferences based on the sequences of individual loci. (**A**). ML phylogenies reconstructed on the *gyrB* locus and (**B**) a concatenated alignment of genes within the seven-loci MLST scheme for *Bacillus cereus*. The adjacent strips represent taxonomic attribution specified in the NCBI Assembly database. The color depicts the species.

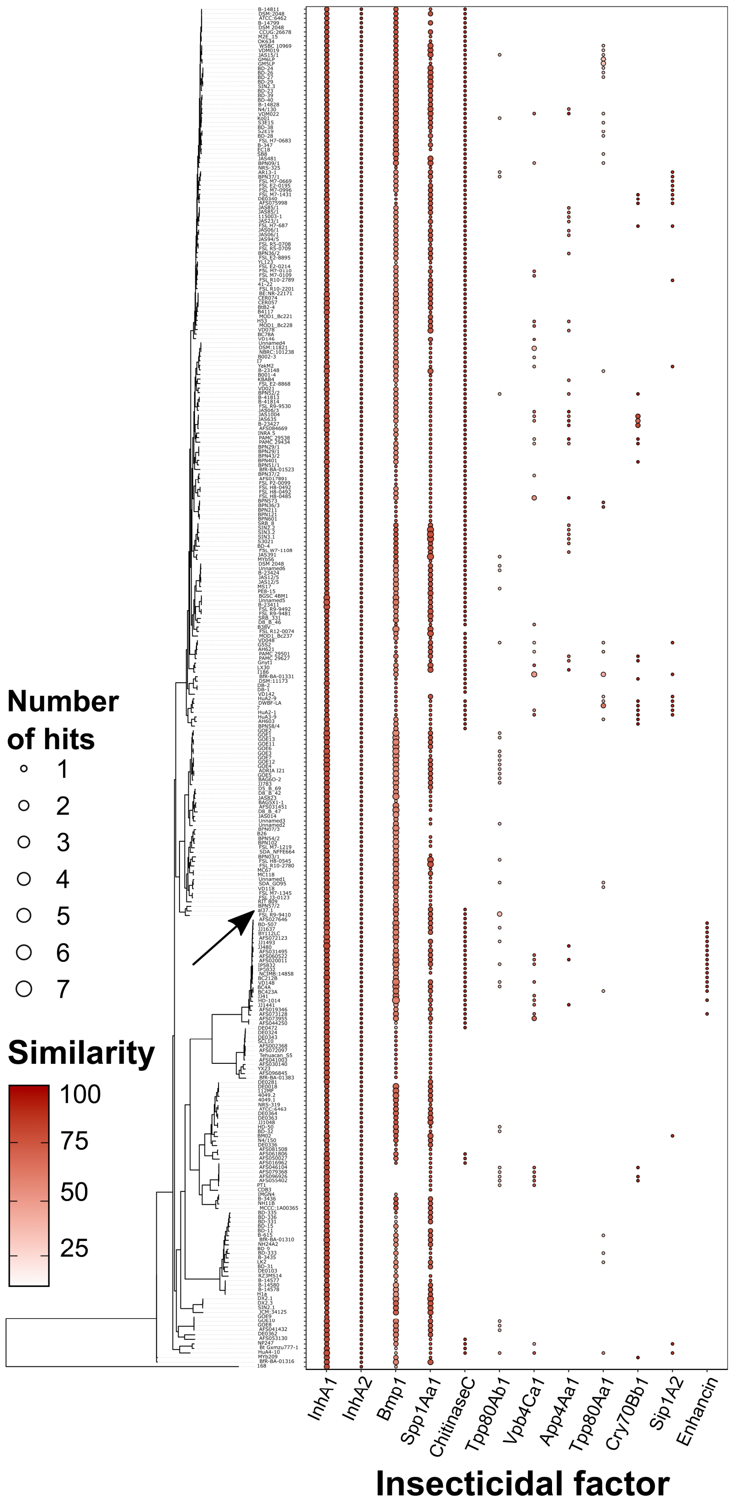

Figure S2. The results of genome mining of insecticidal factors in the genomic dataset. The strain-wise distribution of predicted insecticidal factors. The vertical axis is arranged according to the adjacent ML phylogeny. Plotted on the horizontal axis are individual insecticidal moieties. The size of the dots is proportional to the number of paralogous genes encoding respective factors. The color denotes the mean identity with known homologs from the BPPRC database. Toxins associated with not less than 15 strains are presented. For a full list of inferences, see **Table S6**.

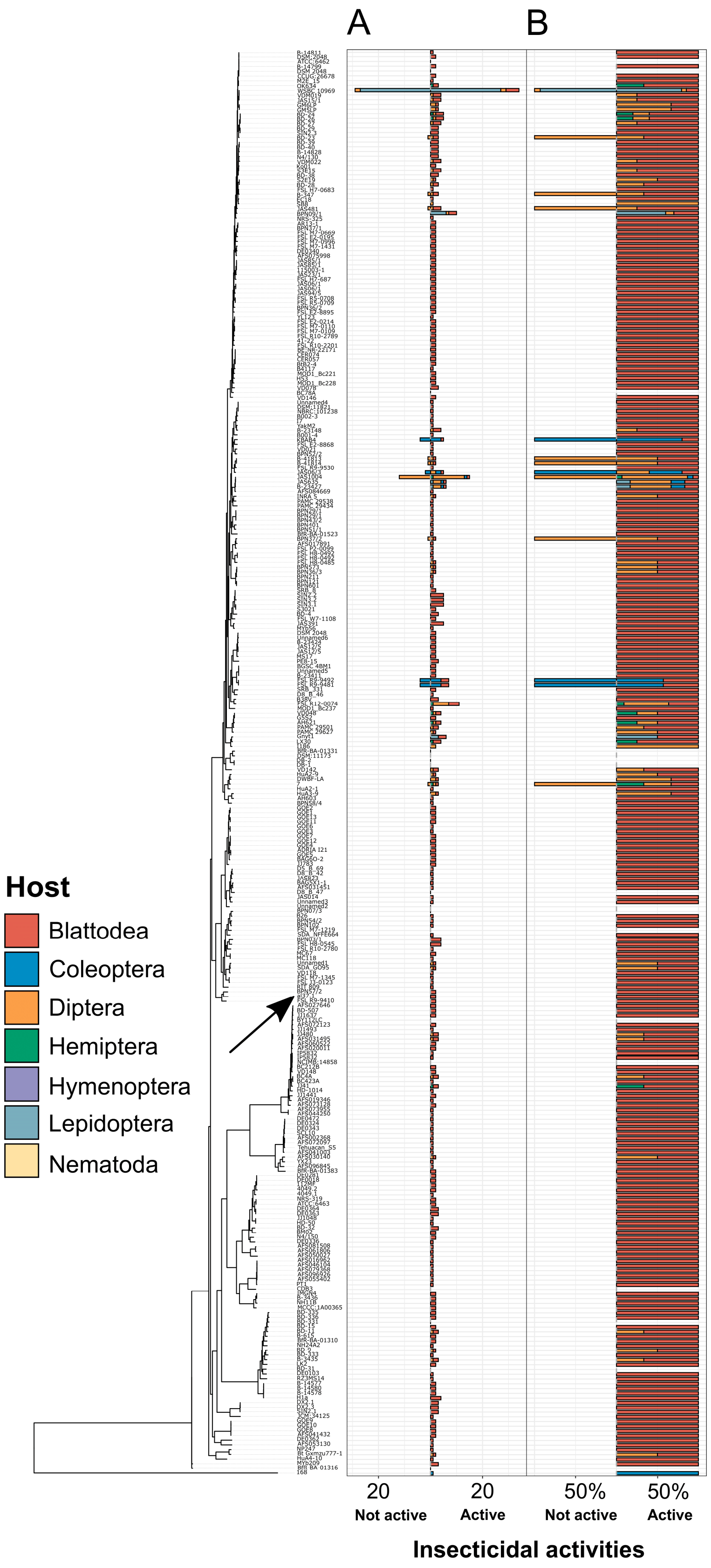

Figure S3. The spectrum of insecticidal properties of the analyzed strains. (**A**) The total number and host-wise proportion (**B**) of predicted activities in the strains according to the sets of insecticidal loci they carry. The specificity data is taken from the BPPRC database. Both positive activities and the absence of toxic effects are displayed. The size of the bars represents the number of individual affected species, coloured according to their orders. The underlying data per strain is presented in **Table S5**.

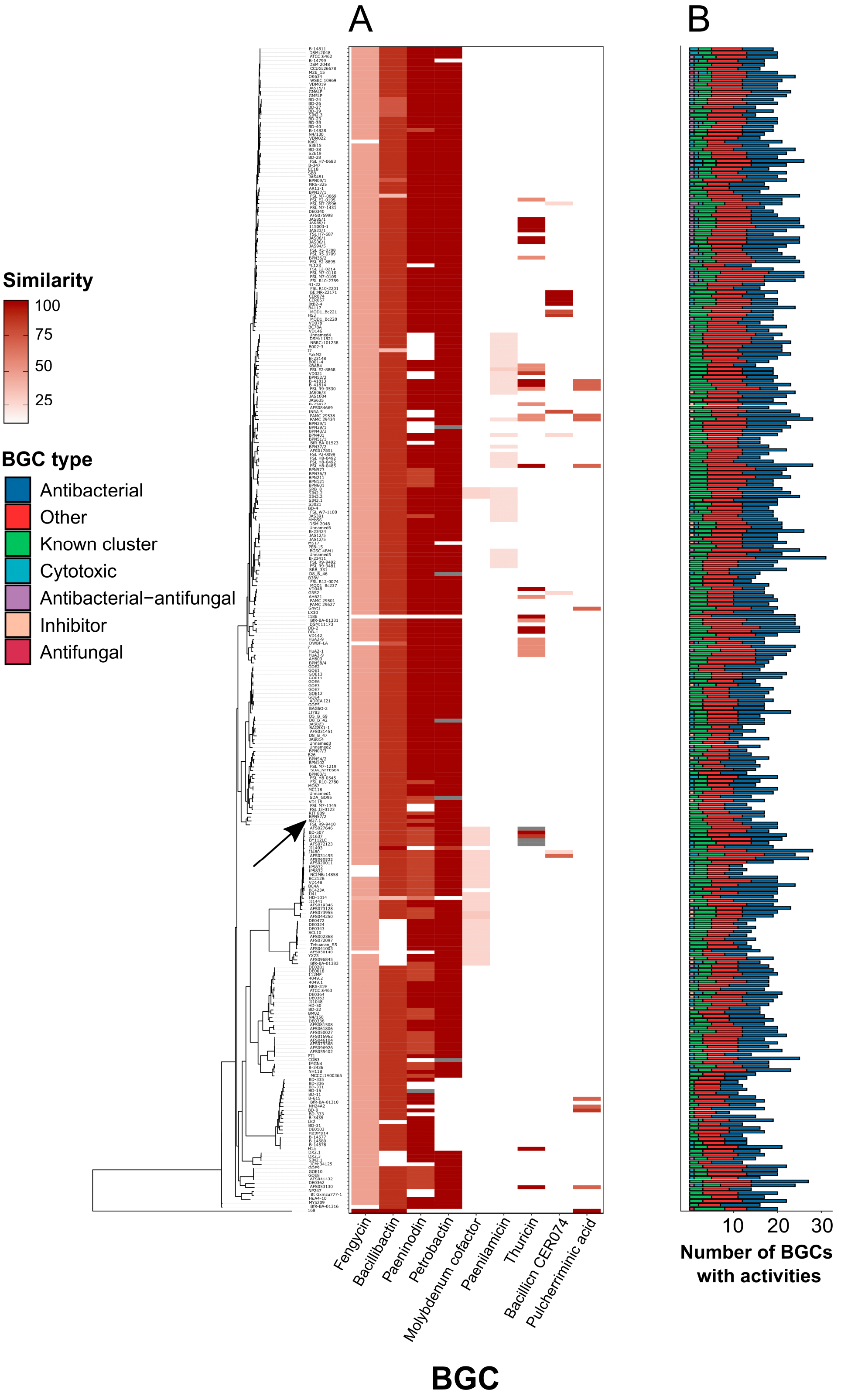

Figure S4. Metabolic potential of the studied isolates in terms of produced BGCs. In all the figures, the vertical axis is arranged according to the adjacent ML phylogeny. (**A**) The spectra of known BGCs identified with the antiSMASH v7.1.1 software. The tiles are coloured according to the mean identity of the core genes with the respective reference BGC. Shown are BGCs found in more than 10 genomes. All hits are available in **Table S7**. (**B**) The total number of BGCs found in the assemblies according to the joint output from antiSMASH and DeepBGC v0.1.30 tools. The BGCs are classified according to the predicted biological activities. The BGCs without putative activities are classified as known or others in case BGCs represent homologs of known clusters or unknown regions, respectively. The exact number of classified BGCs is given in **Table S8**.

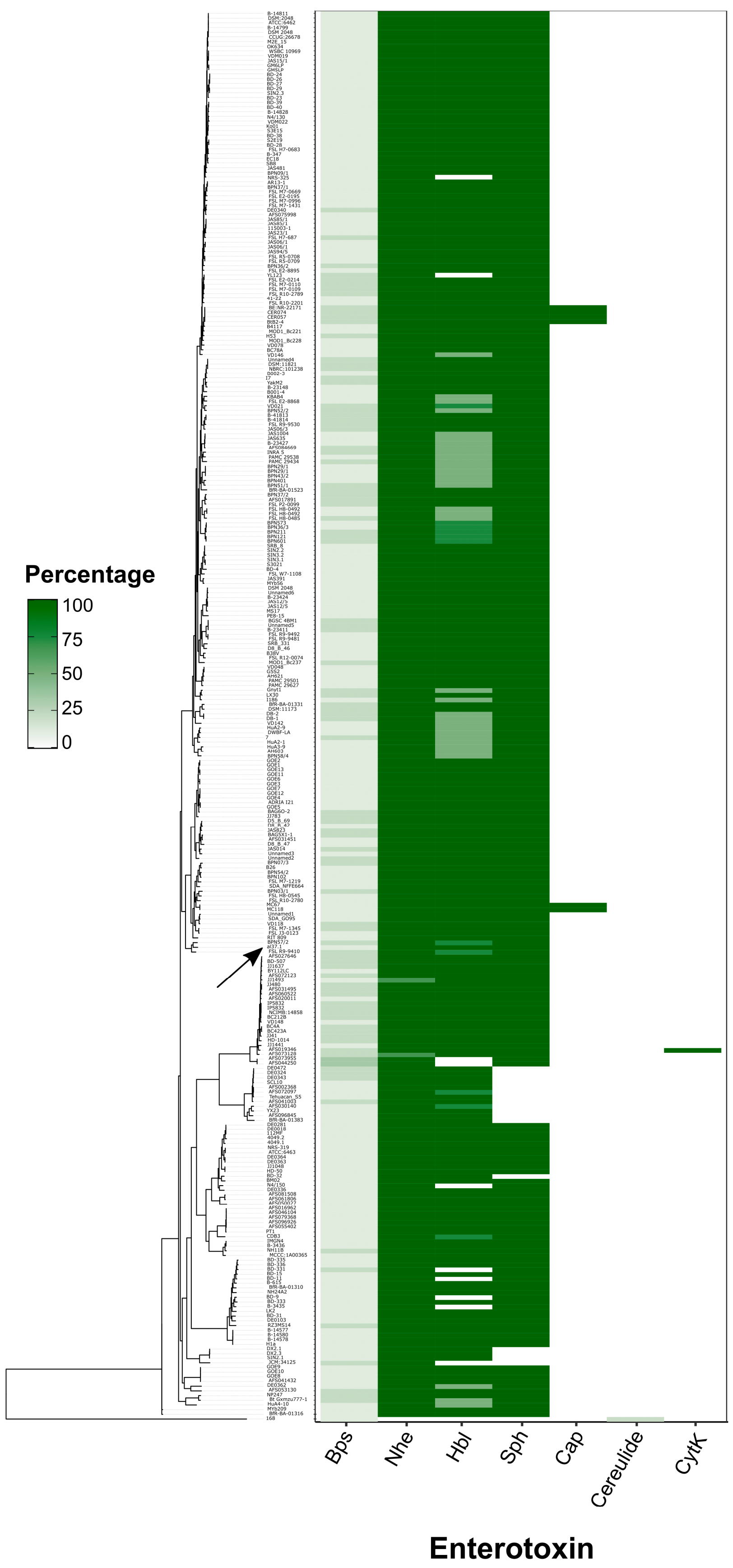

Figure S5. Assessment of the safety of the analyzed strain from the *B. mycoides* group based on predicted toxicity. The presence of enterotoxin-encoding genes identified with the Btyper3 v3.4.0 software. The color of the tiles is proportional to the percentage of genes relative to the reference operon. The vertical axis is arranged according to the adjacent ML phylogeny. Identified genes within the virulence-associated regions is described in **Table S9**.

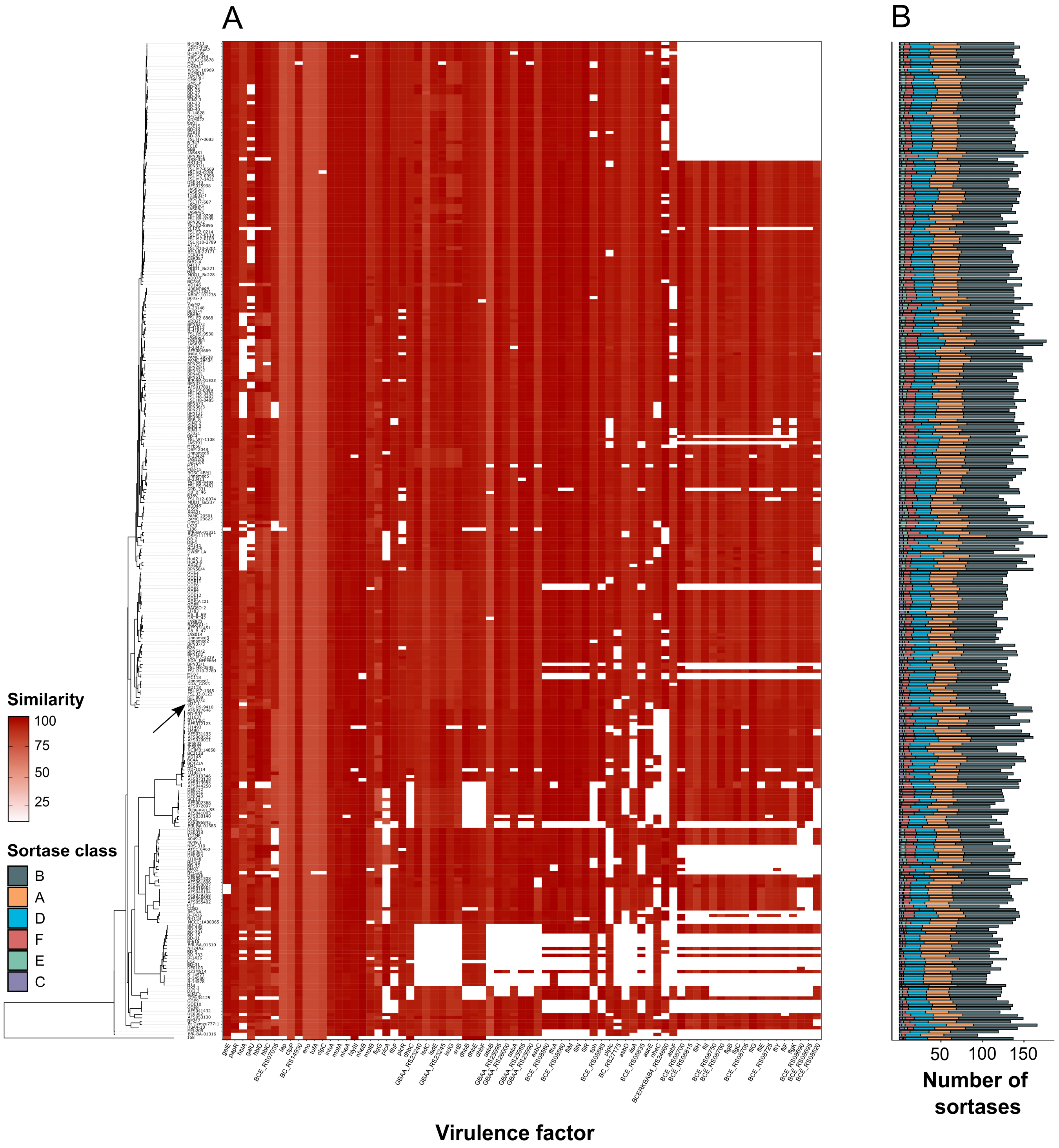

Figure S6. Predicted virulence potential of the strain al37.1^{T} and its closest reference genomes. (**A**) The heatmap illustrating the presence of known virulence determinants from the VFDB resource. The color represents the similarity of protein sequences with the closest reference homolog. Only homologs present in at least half of the genomes are illustrated. The full untrimmed data is presented in **Table S10**. (**B**) The number of sortases produced by the analyzed genomes according to the SortPred v1.0 predictions. Individual sortase classes are colored as the sectors of the barplots. To get the exact values of the identified sortases, see **Table S11**.

2. Supplementary tables description

Table S1. Joint metadata from the NCBI Assembly and BioSample databases for the analyzed genomic dataset. Included are the accession numbers in the respective databases, common descriptions, taxonomic assignments, and quality metrics.

Table S2. Unified taxonomic characterization of the strain al37.1^T coupled with the closest reference genomes. In the table, selected metadata, genomic characteristics, and whole-genome comparisons are given. In the latter case, ANI and DDH values were evaluated relative to the strain al37.1^T ("*ANI_al37_1*", "*DDH_al37_1*"), and the reference *B. mycoides* strain DSM 2048 ("*ANI_DSM_2048*", "*DDH_DSM_2048*") is presented. The rows describing type strains are highlighted with a pink color.

Table S3. The distribution of alleles from the MLST scheme for *Bacillus cereus* as defined in the PubMLST database. The strains are ordered according to the pangenomic phylogeny. The color highlights the suspected species in the *B. mycoides* complex, namely, true *B. mycoides* (green), B. altaicus, and a putative unnamed species (red). The MLST clonal complexes are identified with BTyper3 and mlst software. The alleles for the individual genes were taken from the latter tool.

Table S4. Identity estimates between conserved genes within the MLST scheme for *Bacillus cereus* taken from the PubMLST database. The identity for each gene is calculated between selected type strains and the strains al37.1^T and 2048^T, respectively.

Table S5. The joint characteristics of insecticidal potential within the analyzed dataset of the representatives of the *B. mycoides* species complex. Presented are the identified hits, similarity estimates, mutual coverage, and genomic properties of the respective loci. In case the hit was identified with the HMM method only, the name of the model is given. For each insecticidal moiety, the toxicity data is presented regarding the insects to which these factors are active or not active against.

Table S6. The distribution of the identified insecticidal loci in the analyzed genomes. In case multiple paralogs were identified in the genome, the identity represents the mean estimate.

Table S7. The summary of BGCs (Biosynthetic Gene Clusters) detected in the analyzed strains. Presented are the produced compound and the mean similarity of the core genes with the reference cluster, calculated using the antiSMASH utility.

Table S8. Predicted biological activities of the BGCs in the analyzed genomic dataset. The spectrum of activities was revealed with the DeepBGC software.

Table S9. The distribution of the genes responsible for the synthesis of enteric toxins in the analyzed genomes. The output corresponds to the BTyper3 software used to detect enterotoxins. The detected loci from the operons are listed in parentheses for each virulence factor.

Table S10. The total number of sortases attributed to certain classes according to the SortPred software in the genomic dataset.

Table S11. The spectrum of virulence determinants representing the homologs of the known factors deposited in the VFDB resource.
